# The Two Newsboys

**Published:** 1879-09

**Authors:** 


### THE TWO NEWSBOYS.

"Say, Charley, how much money have you made to day?"

"Twenty-five cents."

"Golly, is that so? Don't tell your mother how much you have made; keep part of it yourself."

The little fellow straightened up, and with great earnestness exclaimed :

"*Do you think I would tell my mother a lie?*"

Turning to the little fellow with an approving smile, I said : "That is right, my boy, always tell the truth."

Noble little fellow! if he abides by that principle of truth he may rise from his humble position to one of usefulness and honor.

